# Detecting and Salvaging Head Impacts with Decoupling Artifacts from Instrumented Mouthguards

**DOI:** 10.1007/s10439-025-03689-z

**Published:** 2025-02-08

**Authors:** Ryan Gellner, Mark T. Begonia, Matthew Wood, Lewis Rockwell, Taylor Geiman, Caitlyn Jung, Blake Gellner, Allison MacMartin, Sophia Manlapit, Steve Rowson

**Affiliations:** 1 Virginia Tech (Biomedical Engineering and Mechanics), Blacksburg, VA USA; 2 Carnegie Mellon (Mechanical Engineering), Pittsburgh, PA USA; 3 Wayne State University (Biomedical Engineering), Detroit, MI USA

**Keywords:** Instrumented mouthguard, Machine learning, Decoupling, Detection, Salvage, Artifact

## Abstract

In response to growing evidence that repetitive head impact exposure and concussions can lead to long-term health consequences, many research studies are attempting to quantify the frequency and severity of head impacts incurred in various sports and occupations. The most popular apparatus for doing so is the instrumented mouthguard (iMG). While these devices hold greater promise of head kinematic accuracy than their helmet-mounted predecessors, data artifacts related to iMG decoupling still plague results. We recreated iMG decoupling artifacts in a laboratory test series using an iMG fit to a dentition mounted in a NOCSAE headform. With these data, we identified time, frequency, and time-frequency features of decoupled head impacts that we used in a machine learning classification algorithm to predict decoupling in six-degree-of-freedom iMG signals. We compared our machine learning algorithm predictions on the laboratory series and 80 video-verified field head acceleration events to several other proprietary and published methods for predicting iMG decoupling. We also present a salvaging method to remove decoupling artifacts from signals and reduce peak resultant error when decoupling is detected. Future researchers should expand these methods using on-field data to further refine and enable prediction of iMG decoupling during live volunteer use. Combining the presented machine learning model and salvaging technique with other published methods, such as infrared proximity sensing, advanced triggering thresholds, and video review, may enable researchers to identify and salvage data with decoupling artifacts that previously would have had to be discarded.

## Introduction

In recent decades, researchers have accumulated evidence about the short- and long-term health implications of repetitive head impact exposure through sport and occupation [1–3]. Most concerning are findings that the length of time playing a contact sport, leading to more time receiving repetitive head impacts, may play a significant role in health outcomes, including the risk of chronic traumatic encephalopathy. In response, many research studies have focused on quantifying head impact exposure in youth and collegiate athletes [4–9]. These studies have begun to converge on using impact monitoring mouthpieces of varying styles, because they couple directly to the skull and provide six-degree-of-freedom kinematics during head impact events.

Rulemaking bodies tie their decisions to head impact exposure data [10–12]. Accurate data are critical for drawing correct conclusions and making the best decisions for athletes. Although many commercially available instrumented mouthpieces have been evaluated or validated in a laboratory environment [13], these devices are subject to error in the field due to effects not present in the lab. Instrumented mouthpieces are in a wet environment when used, constantly subject to wear and tear, and may be in non-ideal positions when an impact occurs. An example of non-ideal positioning could be that the wearer has opened their jaw or has momentarily shifted the mouthpiece away from its coupled position to communicate with teammates. Additionally, mouthpieces may be subject to relative motion during an impact, even if in an ideal position when an impact is initiated. [14–16]

Recently, advanced instrumented mouthguard (iMG) data post-processing techniques to remove data with artifacts or to remove data artifacts themselves have come into the literature. Optimal filters have been suggested based on impact duration, possibly determined by helmet status in sport [17]. Another method, HEADSport, is an openly available iMG data post-processing pipeline developed to create a common and robust method to post-process iMG field data [15]. This method selects the most appropriate filter based on signal frequency content. Another method has begun using artificial intelligence to remove noise from iMG-based kinematic signals [18]. This method showed improvement relative to laboratory reference measurements in tightly coupled iMG signals without additional artifacts and in iMG signals contaminated by lower jaw motion into the mouthguard. Other methods have been suggested that do not use kinematic signals but instead employ teeth proximity sensors within iMGs. These sensors are meant to detect whether the iMG is on or off the teeth and typically have a lower sampling rate than accelerometers or gyroscopes. A method to determine the coupling status of head acceleration events via these proximity readings has been suggested by Luke et al. [19]. Some of these advanced techniques are adaptive to signal content. Others attempt to diagnose iMG decoupling via extra sensors (i.e., teeth proximity sensors). However, no techniques to date attempt to detect or attenuate artifacts specific to iMG decoupling via kinematic signals.

Researchers have previously deployed other automated algorithms to weed out non-impact events from impact events using kinematic signal frequency content, proximity sensor, time, and video-based input features [20–22]. Wu et al. developed a support vector machine algorithm that analyzed frequency-domain data from head impacts measured by iMGs; this algorithm was also informed by the proximity of the iMG to the teeth. In another study, Wu et al. described a machine learning algorithm with more input features, including multi-axial time-domain and frequency-domain data from individual head acceleration events. Gabler et al. used time-domain and power spectral density features to automatically separate impact from non-impact events in American football. Rezaei and Wu described a method of detecting soccer headers from video of soccer matches. The results of these studies were used to reduce the manual processing required to validate head impact data properly [23] and are intended to remove data not representative of true head acceleration events rather than salvage data within head acceleration events.

Previous research has found that decoupling events contain specific time- and frequency-based characteristics when recreated in a laboratory [14]. Our study seeks to develop an algorithm to detect decoupling artifacts and salvage head impact events from field measurements when decoupling is detected. Even when severe decoupling occurs, data from instrumented mouthpieces can contain correct head kinematic data for a reasonable period of the collection window [16]. Usually, this short period is all that is necessary to report accurate peak acceleration values. This research also found specific frequencies associated with decoupling, so applying a logically selected set of filters may enable data salvaging. We do not anticipate every impact will be salvageable. Still, our goal is to increase the ability to keep larger quantities of accurate data in head impact studies, as generating head impact exposure data with volunteers is a time- and resource-intensive process, and rare individual events (i.e., injury) are especially important to get right.

For the purposes of this study, we will refer to head impacts as a real, direct contact to the head that may or may not be measured by a worn iMG or another sensor. A measured head impact will be a direct contact to the head that was measured by a worn iMG or another sensor. A head acceleration event, on the other hand, is a real event that can be caused by direct contact to the head or indirect motion of the head and may or may not be measured by a worn iMG. A measured head acceleration event is captured by a worn iMG or another sensor. Throughout this article, we will call our measured laboratory impacts “head impacts,” because all laboratory impacts involved direct contact to the head. Measured field impacts will generally be referred to as “head acceleration events,” because we do not know that head contact was the only source of head motion for most field events.

## Methods

### Development of a Machine Learning Model for Decoupling Detection

We used data from a laboratory study that explored the effects of decoupling on iMG accuracy to develop a machine learning algorithm that reliably detected head impacts with decoupling event features [14]. We then applied this algorithm to field data from video-verified head acceleration events collected from female adolescent ice hockey players. We compared our algorithm’s outputs to three other impact classification methods—the iMG manufacturer’s change-in-proximity measurement (Delta-prox), the iMG manufacturer’s impact quality label (Quality), and a published method from Luke et al. that uses iMG-specific proximity measurement thresholds. [19]

Our previous study defining decoupling events in the laboratory used a National Operating Committee on Standards for Athletic Equipment (NOCSAE) headform mounted to a Hybrid-III 50th percentile male neck [24] and modified with a mouth area cut out to fit 3D-printed upper teeth and a lower aluminum plate [25] to study the effects of instrumented mouthguard decoupling. This surrogate headform represents the properties of a 50th percentile male [26] and was mounted on a linear slide table with 5 degrees of freedom. A boil-and-bite iMG (Prevent Biometrics, Edina, MN, USA) was fitted to the upper teeth. The fit was confirmed to pass the open-mouth test by pressing the instrumented mouthguard onto the upper dentition and ensuring it did not fall off the teeth. We placed the lower aluminum plate at three distances (0 mm—control, 1.6 mm—small gap, and 4.8 mm—large gap) from the iMG after firmly pressing the iMG onto the upper teeth. The lower plate is attached to the head via a bolt at the midpoint between the rear molars. The lower plate was securely fastened during all tests by tightening it against either the instrumented mouthguard in the coupled conditions or against a series of washers in the open-mouth conditions. These washers effectively tightened the lower plate to the roof of the headform’s mouth without touching the instrumented mouthguard. The lower plate was not free to move during any of the tests.

**Fig. 1:**
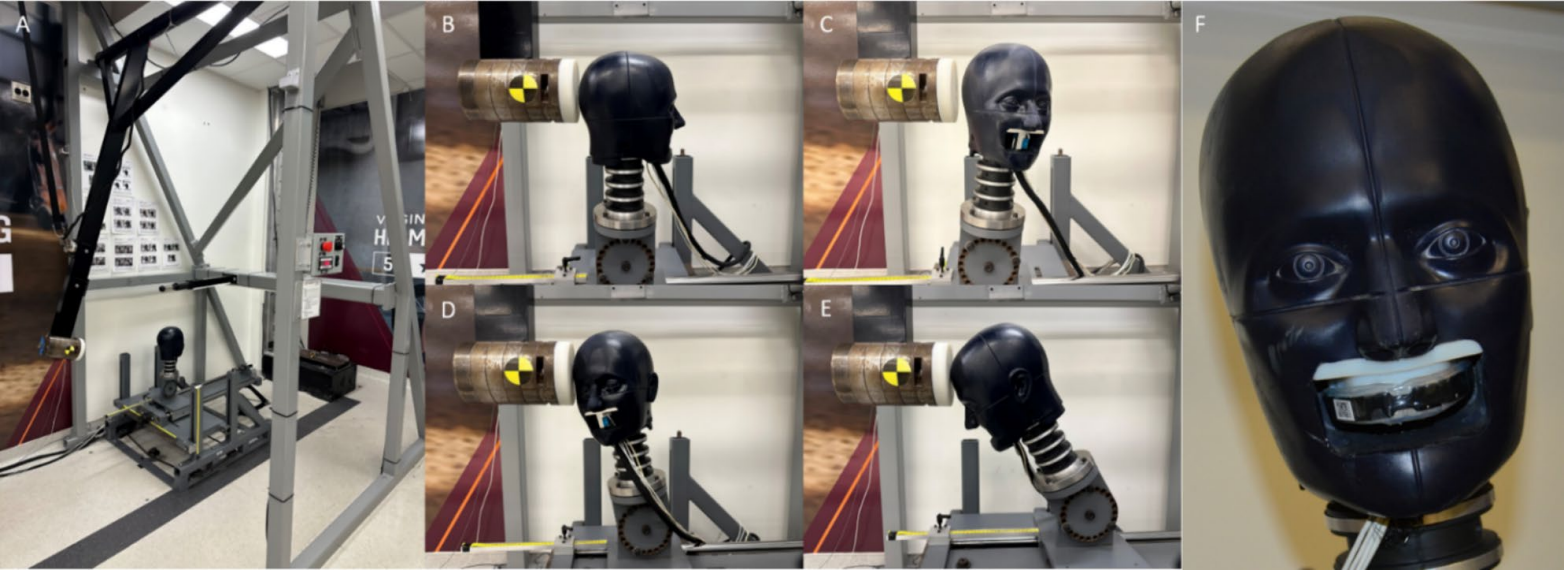
The pendulum impactor **A** setup with four impact locations. **B–E**. A boil-and-bite iMG was fit to 3D-printed teeth in a medium NOCSAE headform with measured gaps to the fixed lower dentition (**F**). We used data from this test series to train the machine learning classification algorithm

We conducted tests on a pendulum impactor at various impact durations and severities (Fig. 1). Two impactor faces (padded (VN600, Dertex Corporation) and rigid nylon), four target peak linear acceleration severities (25, 50, 75, and 100 g), four locations (front, front boss, rear, and rear boss) [25, 27], and two trials at each condition generated 192 total tests. Both impactor faces were 127 mm in diameter; the padded impactor face was 40 mm thick, while the rigid nylon was 25 mm thick. The faces generated linear impact durations in the 3–5 ms range (rigid) and 10–12 ms range (padded). The pendulum arm was 1.905 m long and the anvil mass was 15.5 kg.

The iMG did not capture 32 rigid impacts, leaving 160 valid impacts for input to our machine learning algorithm as training data. Of the valid head impacts in our laboratory training dataset, 104 were with a gap and 56 were in tight conditions (Table 1). We recorded all head impacts with a high-speed camera (Sony Handycam Digital 4 K [FDR-AX700], Sony Corporation, Tokyo, Japan). With these recordings, we were able to determine which impacts had visible iMG motion relative to the teeth and which did not. Of the original 160 impacts, 150 had clear coupling status on video. We found that 37 (25%) of the impacts with a gap condition had clear decoupling from the teeth, 57 (38%) of the impacts with a gap condition did not have decoupling from the teeth and the other 56 (37%) did not have a gap. This left 37 head impacts (25%) with decoupling and 113 (75%) without. We defined decoupling as visible motion of the instrumented mouthguard relative to the headform or dentition, as observed on high-speed video. The high-speed video was captured at 1000 frames per second at approximately 3 feet from the headform with 4-K resolution.

**Table 1:** Laboratory head impacts captured by the iMG by condition and decoupling status.

Impactor face	Gap (mm)	Location	Decoupled	Coupled	Unclear	Total
Pad	0	Front	0	8	0	8
		Front Boss	0	8	0	8
		Rear	0	8	0	8
		Rear Boss	0	8	0	8
	1.6	Front	2	6	0	8
		Front Boss	1	1	6	8
		Rear	3	5	0	8
		Rear Boss	3	4	1	8
	4.8	Front	5	2	1	8
		Front Boss	6	2	0	8
		Rear	4	4	0	8
		Rear Boss	3	4	1	8
Rigid*	0	Front	0	7	0	7
		Front Boss	0	2	0	2
		Rear	0	8	0	8
		Rear Boss	0	7	0	7
	1.6	Front	1	5	0	6
		Front Boss	0	2	0	2
		Rear	0	8	0	8
		Rear Boss	1	3	1	5
	4.8	Front	1	2	0	3
		Front Boss	0	2	0	2
		Rear	2	6	0	8
		Rear Boss	1	5	0	6

*Because the iMG did not collect 32 rigid impacts, the totals for Rigid are less than the totals for Pad

This previously published study showed effects from impact location, with front and front oblique impacts generating greater mean errors relative to the center of gravity (CG) measurements than rear or rear oblique impacts. Results also showed effects from the gap condition, with more significant gaps introducing greater errors. The frequency content of decoupling events differed from coupled impacts, with gap conditions showing higher frequency amplitudes than the tight conditions in the 100–500 Hz range along specific axes of linear (Z-axis) and angular acceleration (X and Y axes). These axes represent the motion of the iMG relative to the teeth during a decoupling event, with the iMG moving inferiorly and rotating in the coronal or sagittal planes. We incorporated these axes and frequency ranges found in our previous study into features for our machine learning algorithm for the current study.

Laboratory data were collected at 20 kHz and filtered at SAE recommended filter classes for impact test instrumentation mounted at the headform CG: three linear accelerometers (Endevco 7264b-2000, 2000 *g*, PCB Piezotronics, Depew, NY) at CFC1000 (cut-off frequency 1650 Hz) and a tri-axial angular rate sensor (DTS ARS3 Pro, 314 rad/s, Diversified Technical Systems, Seal Beach, CA) at CFC180 (cut-off frequency 300 Hz) [28]. Laboratory data collection was triggered when the primary axis for an impact exceeded 5-g linear acceleration; data were collected for 50-ms pre-trigger and 100-ms post-trigger. Instrumented mouthguard instrumentation (three-axis accelerometer, 200 g, and three-axis gyroscope, 35 rad/s) [29] collected data at 3200 Hz and was filtered at previously recommended optimal values for the lowest error relative to laboratory reference data: linear accelerometers at 175 Hz and gyroscopes at 250 Hz [17]. Data collection was triggered for the iMG when any single axis, in the iMG coordinate system, exceeded 8-g linear acceleration; data were collected for 10-ms pre-trigger and 40 ms after the trigger. All signals were re-oriented to match the SAE J211-1 coordinate system [28], gyroscope signals were differentiated using a five-point stencil method to obtain angular acceleration [30], and linear acceleration was combined with angular velocity and angular acceleration according to Equation 1 to generate linear acceleration at the head CG.

1 $$\overrightarrow{{a}_{CG}}=\overrightarrow{{a}_{P}}+\overrightarrow{\omega } \times \left(\overrightarrow{\omega }\times \overrightarrow{r}\right)+\overrightarrow{\alpha }\times \overrightarrow{r}.$$

In Eq. 1, *a*_*i*_ is linear acceleration at point *i*, *r* is the vector distance from point CG to point *P*, *ω* is the angular velocity, and *α* is the angular acceleration of the rigid body.

### Feature Creation and Selection

Based on the results from our previous laboratory study, we generated seven features as an initial feature set for our machine learning algorithm. These features were related to the cumulative frequency content in a given axis between 100 and 500 Hz (Table 2, Features 1–3), the order of the global maximum in the linear acceleration resultant curve (Feature 4), and the time of the maximum amplitude of the frequency content in a given axis, as calculated by a short-time Fourier transform (STFT, Features 5–7).

**Table 2:** List of original features considered for input to the machine learning algorithm

Description	Feature	Axis	Units	Observed range*	Used in final model
Cumulative distribution function of frequency content between 100 and 500 Hz	1	Linear Accel Z	Percent	14.6–73.3	Yes
	2	Angular Accel X		4.86–72.2	No
	3	Angular Accel Y		16.3–68.9	Yes
Order of global maximum peak	4	Linear Accel Resultant	–	1–16	Yes
Time of maximum frequency amplitude	5	Linear Accel Z		0–34.4	No
	6	Angular Accel X	Milliseconds	0–36.3	No
	7	Angular Accel Y		0–36.3	Yes

*Observed range represents laboratory dataset

We calculated the first three features using a fast-Fourier transform (FFT, MATLAB: *fft*), which we multiplied by two and divided by the signal length (2/N). We then took the absolute value and limited the analysis to the Nyquist frequency. We performed zero-padding so that the FFT signal was five times the original length. The value of the cumulative distribution function (CDF) of the FFT at 100 Hz was subtracted from the value of the CDF at 500 Hz. We expected tightly coupled head impacts to have a low magnitude of frequency content between 100 and 500 Hz relative to decoupled head impacts. The fourth feature used MATLAB’s *findpeaks* function to locate all local peaks from the resultant linear acceleration. Any peaks before 0 ms (iMG trigger) were ignored as artifacts and were always smaller than the actual impact peak. We identified the order of the global peak from within the remaining peaks. Based on the coupled impacts in our dataset, we expected well-coupled impacts to have their global peak be one of the first three local peaks, while decoupled impacts would have global peaks much later in the signal. We calculated the fifth through seventh features using the *stft* function in MATLAB. We used twelve-millisecond intervals with 90% overlap to create the time bins for the STFT to strike a balance between time and frequency resolution. We applied a Hamming window to the data. The time of the maximum amplitude corresponded to the time bin, in which the maximum frequency amplitude occurred. The iMG sampling rate was 3200 Hz. We expected tightly coupled head impacts to have their maximum frequency amplitude in the 3–15 ms range, while decoupled impacts would have their maximum frequency amplitude later in the signal [14].

The classification label for each impact was either coupled or decoupled. We used the coupling status from our laboratory dataset high-speed video review to label the impacts in our training dataset for training the machine learning model. We observed varying degrees of decoupling in the 1.6- and 4.8-mm gap condition head impacts during testing, representing a range of decoupling features the algorithm would need to classify correctly. We used MATLAB’s Classification Learner app to test different machine learning algorithms on our dataset, with tenfold cross-validation accuracy as the discriminator. The Classification Learner app allows for training ten different types of machine learner algorithms to compare their accuracy on input data (Decision Trees, Discriminant Analysis, Logistic Regression classifier, Naïve Bayes classifier, Support Vector Machine, Efficiently Trained Linear classifier, Nearest Neighbor classifier, Kernel Approximation classifiers, Ensemble classifiers, and Neural Network classifiers). We trained each of the different types offered by the app. The Nearest Neighbor algorithm came out as having the greatest accuracy in training.

Next, we employed the MATLAB experiment manager to iterate through 100 combinations of hyperparameters for the Nearest Neighbor algorithm. We varied the number of neighbors (2–30), the distance calculation method (Manhattan city block, Euclidean, Minkowski, cosine, Chebyshev, Correlation, Hamming, Jaccard, Mahalanobis, Standardized Euclidean, and Spearman), and whether the data were standardized or not. We ranked the algorithms by accuracy in tenfold cross-validation using the entire available dataset. We preferred standardized models with the simplest distance calculation metrics to avoid over-fitting while maintaining accuracy. Additionally, we used the Classification Learner app’s Feature Selection tool to remove less critical features. We used the two ranking systems relevant to our input data containing continuous and ordinal data: Minimum-Redundancy-Maximum-Relevance and Chi-Squared. We recorded the ranking of each feature from each system and summed them. The summed rank from the two feature ranking systems became our ranked order of feature importance. To quantitatively assess the machine learning model’s accuracy, we used the truth data to generate a model confusion matrix (Table 3).

**Table 3:** Exemplar confusion matrix for machine learning model assessment

		Label	
		Decoupling	No decoupling
Model predicted	Decoupling	True positive	False positive
	No decoupling	False negative	True negative

We tried our classification algorithm on field data from the same brand of iMGs. We collected and video-verified head acceleration events from high school female ice hockey players wearing instrumented boil-and-bite mouthguards during an entire season in the field (130 practice or game sessions). All sessions were video recorded, and all head acceleration events greater than 10-g resultant linear acceleration within session times were video-validated by trained research staff. Only head acceleration events reported by the iMG manufacturer as being above the 10-g threshold were considered. However, after identifying the valid head acceleration events, the raw data were used to appropriately post-process [17] and generate 6DOF head kinematic data in the SAE J211 coordinate system at the head CG for each impact, resulting in some head acceleration events having their peak values altered.

We used video-validated iMG-measured head acceleration events. We visually inspected each impact’s kinematic traces to ensure no pre-trigger peaks were included. This visual inspection was meant to eliminate head acceleration events that could have occurred outside the iMG’s reset window for collecting a new impact (e.g., a second impact occurring immediately after the primary) [31]. We used our trained machine learning algorithm to predict whether decoupling had occurred in the remaining 80 field head acceleration events.

### Comparison of Machine Learning Model Predictions to Other Prediction Methods

We compared our machine learning algorithm’s predictions to predictions from three other methods using iMG data from field and lab settings. The first supplementary prediction method was the iMG manufacturer’s Delta-prox measurement. The iMG includes an infrared proximity sensor approximating the distance between the iMG and teeth. The difference between the last proximity reading before an impact and the first reading after an impact is used to determine the change in proximity. If the Delta-prox measurement exceeds a pre-set number of proximity units, the manufacturer considers this impact invalid and does not display it as a true impact in their app. Similarly, we considered head acceleration events that crossed this threshold of proximity units in Delta-prox as predicting a decoupled impact for this prediction method, with anything less than the threshold being a coupled prediction. The second supplementary prediction method was the quality label, which was also output by the iMG manufacturer. Quality is output by the manufacturer’s proprietary algorithm that uses signal features and proximity data to classify head acceleration events into one of three categories: zero, one, or two. Zero-quality head acceleration events are treated as the best and filtered using a corner frequency of 200 Hz. One- and two-quality head acceleration events undergo additional filtering (1: 100-Hz corner frequency, 2: 50-Hz corner frequency) to smooth artifacts from the signal [32]. We treated zero-quality head acceleration events as coupled and one- and two-quality head acceleration events as decoupled.

The third supplementary prediction method was Luke et al.’s method [19]. Luke’s study also employed the iMG’s proximity readings, but rather than using a blanket threshold for change in proximity, they developed a unique threshold for each iMG’s pre- and post-impact proximity readings. They generated a k-means cluster for each iMG, using all of the proximity readings from a given iMG, with an expectation of two clusters and a minimum silhouette score of 0.9. They labeled iMGs with silhouette scores below 0.9 as having an “unclear” coupling status.

The mid-way points between the two cluster means for each iMG was then considered the threshold for pre- and post-impact proximity readings. Therefore, the pre- and post-impact readings could each be in one of two states: coupled (above the iMG’s unique threshold) or decoupled (below the threshold). This labeling method creates four possible predictions for any given impact: decoupled (both readings below threshold), recoupling (pre-impact below, post-impact above threshold), decoupling (pre-impact above, post-impact below threshold), or coupled (both readings above threshold). As Luke et al. did, we considered all but the coupled category as being decoupled.

We modified Luke’s method to characterize our lab data. We used only the pre-, mid-, and post-impact proximity readings to create the cluster means rather than all proximity readings available for the entire proximity reading window (up to 20 s pre-impact). This modification ensured the proximity readings we used were not biased toward the on-teeth value, because our iMG did not move on the teeth for the time leading up to the impact, as it can and often does in the field. Including all the pre-impact proximity data would have resulted in a proximity dataset heavily biased toward on-teeth readings. This biased dataset would have had very few off-teeth readings because proximity recording stops within 100 ms after the impact, and the silhouette score would have suffered because two means would have been difficult to identify with such a biased dataset.

Field iMG data were processed using Luke’s method without modification. Our final field dataset included 80 head acceleration events from 24 unique iMGs. Seventeen iMGs met the silhouette score requirements for Luke et al.’s method, resulting in us dropping 26 of the 80 field head acceleration events for Luke’s method. We compared Luke et al.’s method to the other methods using 54 field head acceleration events rather than 80.

### Salvaging Decoupled Head Impacts

We created a method to salvage data for all model-predicted decoupling events. We applied the salvaging methods to raw iMG sensor data (three accelerometers, three gyroscopes) after scaling to engineering units. Each individual sensor signal was considered a separate input signal, with a corresponding salvaged output signal. Typical post-processing took place after salvaging the individual raw channels. The difference between unsalvaged data and salvaged data is that only a pre-set filter class combination (175 Hz for linear accelerometers, 250 Hz for gyroscopes) was applied via a fourth-order phaseless low-pass filter for unsalvaged data [14]. Salvaged data had the same Butterworth filter applied and was then processed using the wavelet deconstruction methodology described below.

Based on known frequency content from head impact events recorded using iMGs in a laboratory [14, 19–21], we set up a wavelet deconstruction analysis by dividing the input signal into frequency sub-bands. We used MATLAB’s *modwt* and *modwtmra* to decompose each decoupled impact into five detail bands and one approximation band (Table 4). We selected the ‘db10’ (Daubechies wavelet, number 10) with 5 levels. These levels were then selectively kept or rejected based on pre-defined criteria.

**Table 4:** Wavelet deconstruction frequency sub-bands used in the salvaging process

Decomposition level	Frequency band (Hz)
Detail 1	800–1600
Detail 2	399–802
Detail 3	199–401
Detail 4	99.8–201
Detail 5	54.3–101
Approximation	0–45

When decoupling was identified, we applied the wavelet decomposition to filtered, untransformed sensor data from the iMG signals. Based on previous knowledge of coupled head impacts, we rejected detail levels 1 and 2 for all head impacts, and we kept the Approximation. The selection criteria for detail levels 3 through 5 included breaking each level’s time signal down into 10-ms sections. We computed the relative energy for each section by dividing the signal energy in the section by the total signal energy for that level (Eq. 2).

2 $${E}_{r}= \frac{{\sum }_{n=1}^{{L}_{s}}({{x}_{n})}^{2}}{\sum_{i=1}^{L}({{x}_{i})}^{2}}.$$

In Eq. 2, *E*_*r*_ is the relative signal energy for a given decomposition level, *x*_*n*_ is the point *n* in the current section, and *x*_*i*_ is the *i*th point in the full decomposition level’s signal. Note that while* L* is the length of the input signal, *L*_*S*_ is the length of each section, equivalent to the number of samples in 10 ms of data.

**Fig. 2:**
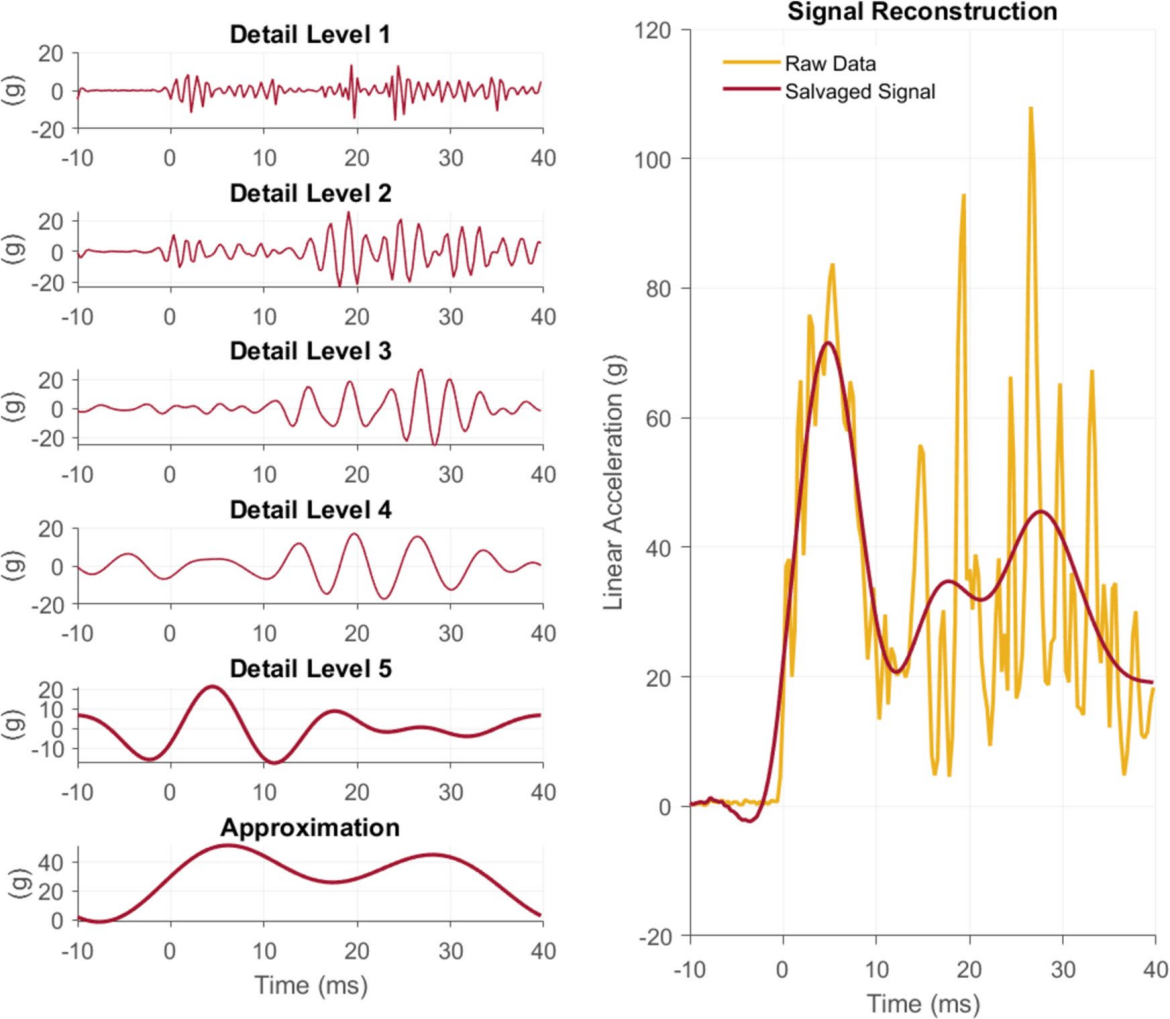
The raw signal was decomposed into six frequency sub-bands (left column). Detail levels 3 through 5 were selected or rejected based on energy content in 10-ms sections. In this linear acceleration signal, levels 3 and 4 were rejected, because the energy in the latter portions was greater than the energy in the primary (0–10) section. The reconstructed signal is shown on the right in maroon compared to the raw data with noise artifacts in orange

For linear acceleration, we compared the first post-trigger (0–10 ms) section’s relative energy to the relative energy in subsequent (10–20 ms and 20–30 ms) sections. If the later grids’ relative energies were higher than the first grid by more than 10% relative energy, we considered that level indicative of decoupling noise and removed it from the salvaged impact. For angular velocity signals, we modified the criteria slightly. First, we removed detail level 3, because angular velocity signals typically have lower frequency than acceleration signals [28]. Additionally, the primary section used for angular velocity signals was 10–20 ms rather than 0–10 ms, because angular velocity signals tend to peak later in coupled head impacts and would therefore be expected to have highest energy in the 10–20-ms grid (see example head impacts, Appendix A). Finally, if level 3 or 4 contained more than ten percent relative signal energy, we rejected it outright, because none of our well-coupled head acceleration events had this relative amount of energy in levels 3 or 4. We reconstructed the salvaged head impacts with the remaining levels at the end of the selection criteria process (Fig. 2).

When decomposing signals using wavelet multiresolution functions, each level contains specific frequency components. These frequency components may exist in the pre-trigger region of the signal, but upon reconstruction will always add to near zero (red signal on right side of Fig. 2). However, the pre-trigger region may not be reconstructed to near zero when we remove certain detail levels entirely. We chose to deal with these edge effects by performing two steps. First, we padded the signals on both ends before performing the wavelet decomposition. We removed the padding before outputting the final reconstructed signal. Second, we included the pre-trigger (− 10 to 0 ms) portion of all detail levels in our final reconstruction. This ensured the pre-trigger region summed to near zero, as it did in the raw data, regardless of which detail levels were selected by our salvaging criteria.

We assessed the accuracy of our methods by comparing peak data from reconstructed laboratory data to peak CG measurements. Percent error was calculated according to Eq. 3. We checked our salvaging process on coupled head impacts to quantify the potential effect on signal accuracy in the case of false-positive decoupling classification from our machine learning model; however, the salvaging process is not meant to be used on coupled head impacts. Finally, we used the salvaging method on all field head acceleration events predicted to be decoupled and quantified the change from the initially reported kinematics in these head acceleration events using Equation 3 representing percent *change* rather than percent *error*, because the ground truth for field measurements was not known. After salvaging, we ran all decoupled, salvaged head impacts from the lab dataset and decoupled, salvaged head acceleration events from the field dataset back through the machine learning algorithm to determine if the salvaging process had successfully converted the prediction to coupled.

2 $${\epsilon }_{\%}= \frac{{PK}_{S} - {PK}_{CG}}{{PK}_{CG}}\times 100\%.$$

In Eq. 3, *PK*_*S*_ is the salvaged peak kinematic value, *PK*_*CG*_ is the peak kinematic value at the center of gravity (laboratory data) or the original (field data), and ${{\varvec{\upepsilon}}}_{\%}$ is the percent error (laboratory) or percent change (field).

We completed all data post-processing in MATLAB R2023b (MathWorks—Natwick, MA) and used RStudio 2023.06.0 (R Foundation for Statistical Computing, Vienna, Austria) to calculate summary statistics and generate plots. The Virginia Tech Institutional Review Board (IRB) approved the field data collection under protocol 21-500.

## Results

### Machine Learning Model for Decoupling Detection

Multiple combinations of hyperparameters in our K-Nearest Neighbor iteration experiment resulted in similar (>90%) accuracy. Options that did not overfit our data were preferred. We selected a simple and robust implementation of the Nearest Neighbor algorithm to ensure our model was generalizable to other data sources, especially field data where inherent signal noise can be greater than in the laboratory. We settled on a medium number of neighbors (4), a simple distance calculation metric (Manhattan city block) with equal weighting distance, and we standardized the data before calculation to avoid overemphasizing features with larger numerical range. Features 2, 5, and 6 were the bottom three features in our feature ranking, so we removed them one at a time. We were able to remove each of these features without sacrificing accuracy, maintaining cross-validation accuracy above 90% in training. Our final model used Features 1, 3, 4, and 7.

Our selected model correctly classified 137 training head impacts (91.3%) across the ten cross-validation folds (Table 5). The model’s sensitivity to decoupling events was 86.5% and its specificity for detecting coupled head impacts was 92.9%.

**Table 5:** Confusion matrix for machine learning model assessment

	Label	
	Decoupled	Coupled
Model predicted		
Decoupled	32	8
Coupled	5	105

### Comparing Machine Learning Model Predictions to Other Prediction Methods

#### Laboratory Data

The three supplementary prediction methods had various levels of predictive capability on our laboratory dataset (Tables 6 and 7). The Delta-prox method correctly identified 100% of the coupled head impacts as such but also incorrectly labeled 70% of decoupled head impacts. The Quality prediction method correctly identified 65 (58%) coupled head impacts, but only 23 (62%) decoupled head impacts. Lastly, the modified Luke et al. method performed similarly to delta-prox., correctly labeling 100% of coupled head impacts but only 30% of decoupled head impacts.

**Table 6:** Summary of method performance in predicting laboratory impact decoupling status

Method	Accuracy (%)	Sensitivity (%)*	Specificity (%)*
Machine learning model	91.3	86.5	92.9
Modified Luke et al.	82.7	29.7	100.0
Delta-prox.	82.7	29.7	100.0
Quality	58.7	62.2	57.5

*A true positive (Sensitivity) is a properly detected decoupling event, while a true negative (Specificity) is a properly detected coupled event

**Table 7:** Laboratory impact predictions by ground truth status

Method	Prediction	Truth coupled	Truth decoupled
Machine learning model*	Coupled	105	5
Modified Luke et al.	Coupled	113	26
Delta-prox.	Coupled	113	26
Quality	Coupled	65	14
Machine learning model*	Decoupled	8	32
Modified Luke et al.	Decoupled	0	11
Delta-prox.	Decoupled	0	11
Quality	Decoupled	48	23

*Machine learning model results from tenfold cross-validation, repeated from Table 4

#### Field Data

We also passed field data through our machine learning algorithm and the supplementary prediction methods. We could not determine whether iMG decoupling occurred in the field head acceleration events, as we recorded video at a wide angle to capture as much of the ice surface as possible during data collection. Thus, the presented predictions are compared to one another but not to a ground truth.

Seventeen of the 24 unique iMGs met Luke et al.’s minimum silhouette score, which left 54 of the 80 field head acceleration events for comparison to other methods. The coupling status was “unclear” for the remaining 26 head acceleration events, according to Luke et al.’s method. For Luke et al., we report the comparisons below with “unclear” as a third category, besides coupled or decoupled.

**Fig. 3:**
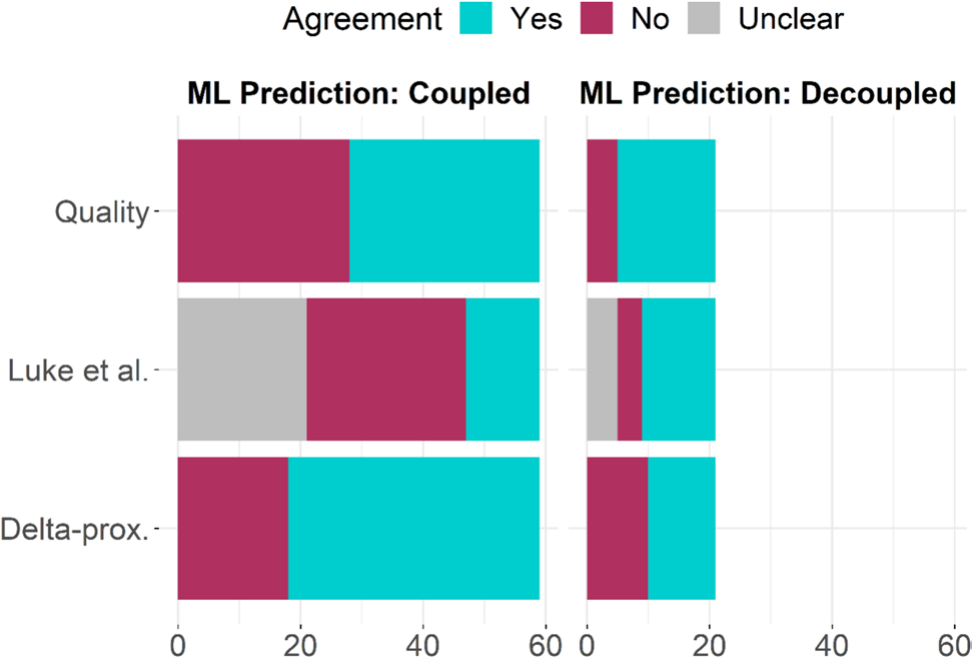
Agreement between prediction methods by the number of field head acceleration events. When the machine learner predicted decoupling in a field impact, Quality was the most likely to agree, followed by Luke et al.’s method. When the model predicted coupled, Delta-prox. was most likely to agree

The developed machine learning model predicted 21 head acceleration events to have decoupled and 59 head acceleration events to be well coupled. The Delta-prox method labeled 29 head acceleration events as decoupled and 51 as coupled. The Quality method predicted 44 head acceleration events as decoupled and 36 as coupled. Finally, the Luke et al. method predicted decoupling in 38 head acceleration events, labeled 16 as coupled, and left 26 as unclear. Figure 3 summarizes the predicted status of each impact with comparisons between methods.

Our machine learning model agreed with the Quality method in 47 head acceleration events (59%). When the model predicted decoupled, the Quality method agreed 76% of the time. Conversely, when the model predicted coupled, Quality agreed in 53% of the head acceleration events. The Quality method predicted decoupling in 59% of field head acceleration events overall, higher than the machine learning model’s decoupling prediction rate of 26%.

When comparing our model to the Luke et al. method, we will discuss without Luke’s “unclear” labeled head acceleration events. Our model agreed with the Luke et al. method in 24 head acceleration events (44% of the head acceleration events with clear coupling status). When the model predicted decoupled, Luke et al. agreed in 75% of head acceleration events, but only 32% of head acceleration events when the model predicted coupled. Ignoring “unclear” head acceleration events, Luke et al. predicted decoupling in 70% of head acceleration events, which was higher than our model’s decoupling prediction rate (26%) in this dataset. The “unclear” head acceleration events from Luke et al. were more likely to be labeled coupled (21 head acceleration events) by our model than decoupled (5 head acceleration events).

Finally, our machine learning model agreed with Delta-prox in 52 (65%) of the field head acceleration events. The other 28 head acceleration events were predicted to be decoupled by the machine learning model but coupled by the Delta-prox method. Delta-prox generally predicted head acceleration events to be coupled more often than decoupled, which was similar to the machine learning model. When the machine learning model predicted coupled, Delta-prox agreed 70% of the time but agreed on only about half (52%) of the head acceleration events when our model predicted decoupled.

We also compared the supplementary methods to one another as a secondary analysis. The Delta-prox. method agreed with the Quality method in 51 head acceleration events, or 64% of field data. As mentioned, Delta-prox. was biased toward predicting a coupled status, while Quality was more balanced between coupled and decoupled. Thus, when Quality predicted decoupled, Delta-prox. only agreed half the time. When Quality predicted coupled, Delta-prox. agreed much more often (29 head acceleration events, 81%).

Luke et al.’s method had a similar overall agreement with Delta-prox. (70%) and Quality (69%) in head acceleration events that were not “unclear.” Head acceleration events that were labeled “unclear” by Luke were biased toward being labeled as coupled by Delta-prox (23 coupled vs. 3 decoupled). On the other hand, Quality predicted slightly more “unclear” head acceleration events as being coupled (17) as opposed to decoupled (9).

### Salvaging Decoupled Data

#### Laboratory Data

**Fig. 4:**
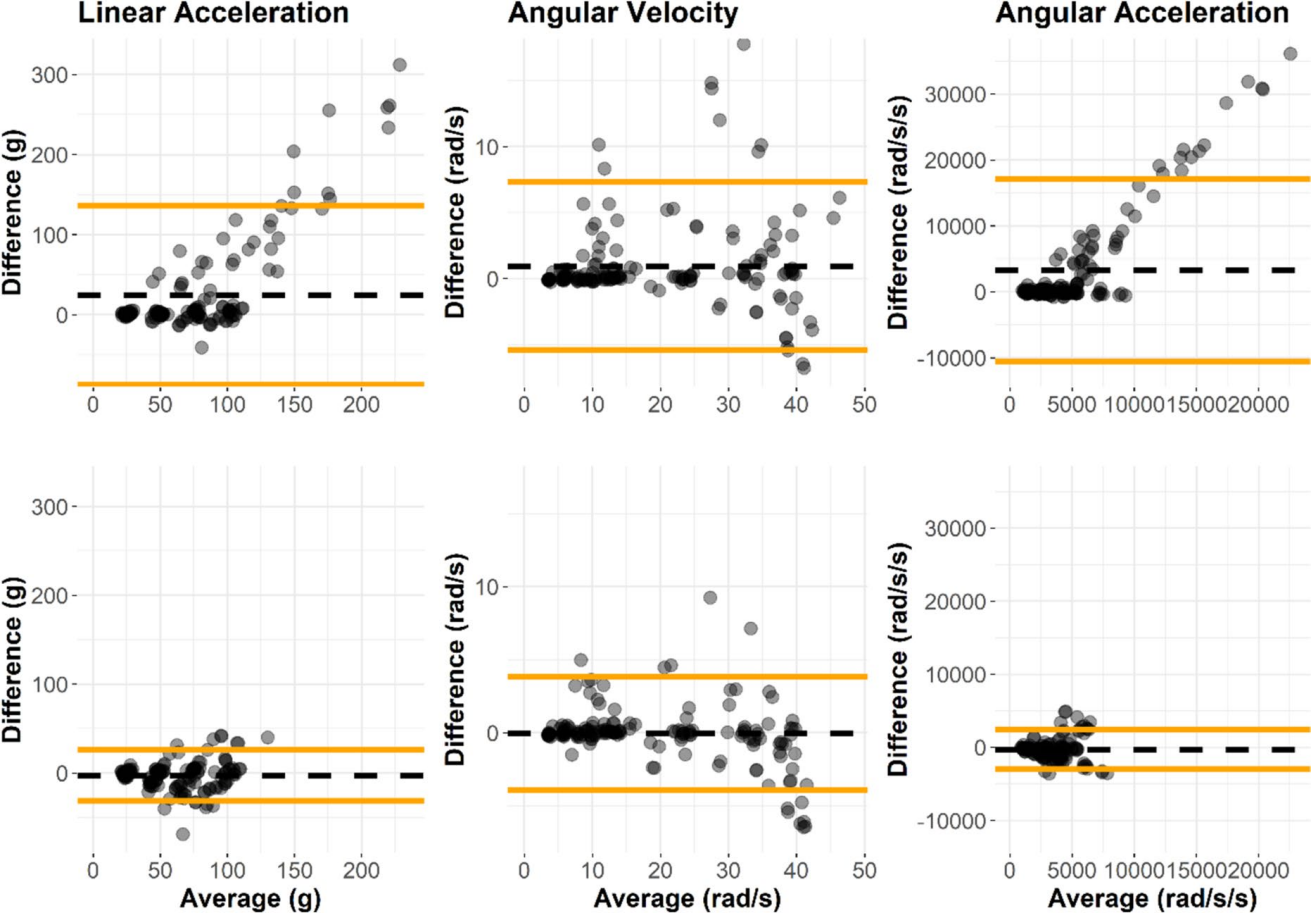
Bland–Altman plots comparing typical pre-salvaged (top row) and post-salvaging (bottom row) peak kinematic magnitudes to laboratory reference measurements in decoupled laboratory iMG head impacts. The salvaging method decreased peak magnitudes and error relative to CG measurements

We applied our salvaging methods to all laboratory-collected iMG impacts. The salvaging process improved accuracy and reduced variance for each of the kinematic measures in decoupled impact events, relative to the reference headform data (Table 8 and Fig. 4). We applied the salvaging process to coupled head impacts solely to understand the effect of the process in case of misclassification by the machine learning algorithm. Minimal effect on peak kinematic values was observed for coupled events.

**Table 8:** Salvaging method effects on laboratory img peak kinematic percent error relative to headform reference peak

Coupling status	Kinematic	Technique	Mean percent error (%)	Standard deviation percent error (%)
Decoupled	Linear accel	Typical post-processing	121	138
		Salvaging process	− 0.31	24.2
	Angular velocity	Typical post-processing	17.6	25.4
		Salvaging process	5.20	17.8
	Angular accel	Typical post-processing	308	296
		Salvaging process	12.6	56.0
Coupled*	Linear accel	Typical post-processing	7.83	35.0
		Salvaging process	− 6.13	17.4
	Angular velocity	Typical post-processing	4.09	21.0
		Salvaging process	1.26	10.3
	Angular accel	Typical post-processing	23.0	90.0
		Salvaging process	− 11.3	36.8

*Coupled head impacts were salvaged to understand effects in the case of misclassification; we do not recommend applying the salvaging method to coupled head impacts

We applied a student’s *t* test to check for differences in distributions of peak magnitudes before and after salvaging. The peak values differed for decoupled impacts in linear acceleration (p < 0.001, mean difference estimate 85.9 g) and angular acceleration (p < 0.001, mean difference estimate 10975 rad/s^2^). Angular velocity peak magnitudes did not differ from before to after salvaging in decoupled head impacts (p = 0.24, mean difference estimate 2.94 rad/s).

Coupled head impacts did not differ in linear acceleration peak (p = 0.39, mean difference estimate 7.4 g) or angular velocity peak (p = 0.86, mean difference estimate 0.24 rad/s) values. Angular acceleration peaks did differ for coupled head impacts (p < 0.001, mean difference estimate 1005 rad/s^2^).

We ran the decoupled, salvaged laboratory head impacts back through the machine learning algorithm to determine if the salvaging process had been successful. In 29 (78%) impacts that had originally been labeled decoupled, the salvaging process successfully converted the prediction to coupled. The remaining 8 (22%) were considered unsalvageable.

#### Field Data

**Fig. 5:**
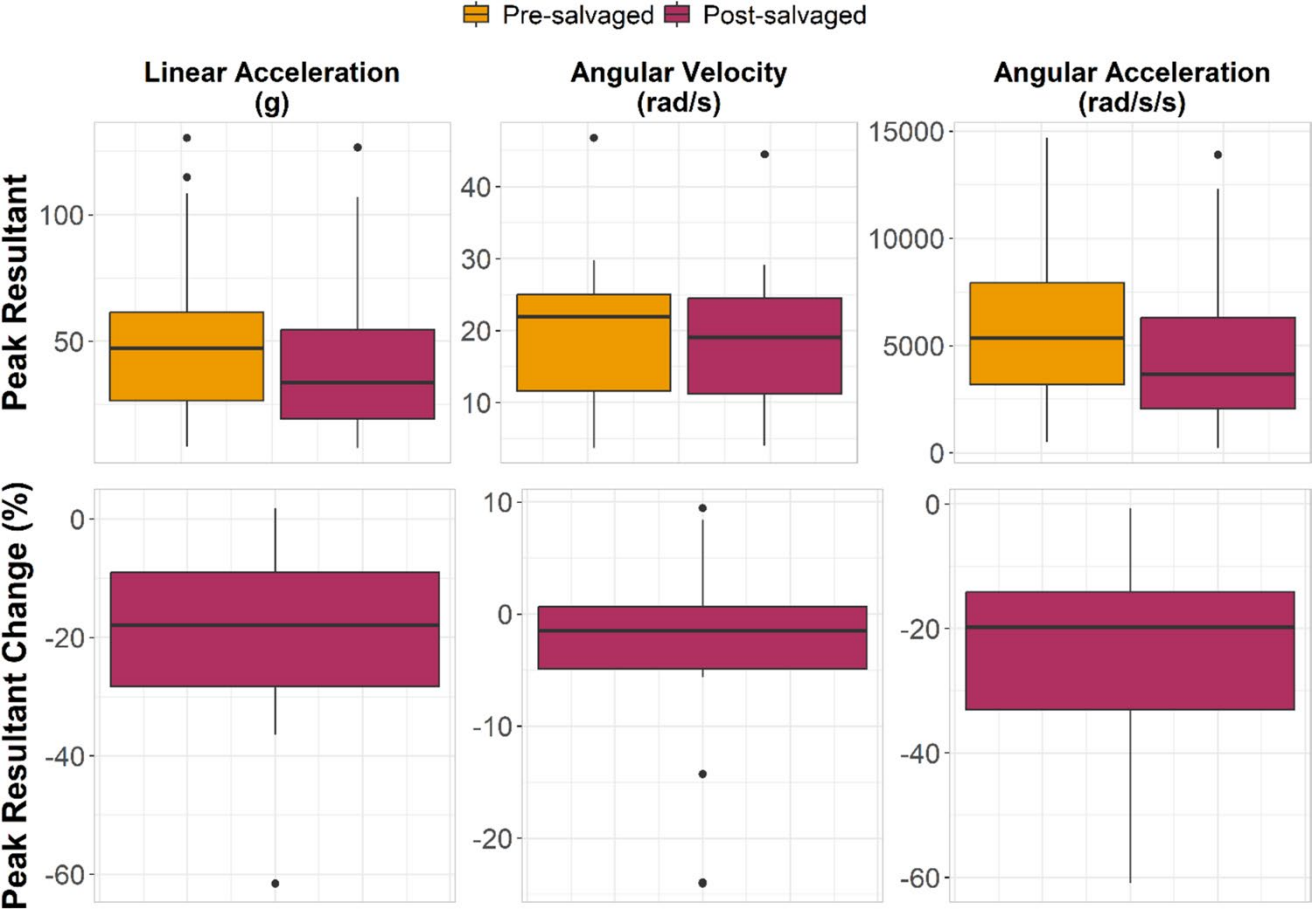
When we applied our salvaging process, field head acceleration events predicted to be decoupled had their peak accelerations decreased by approximately 20% and their peak angular velocity decreased by 3%

We also performed predictions on field head acceleration events and then ran any predicted-decoupled head acceleration events through the salvaging process. The percent change in peak kinematic values for decoupled head acceleration events was − 19.1 ± 14.6% for linear acceleration, − 3.17 ± 8.56% for angular velocity, and − 23.3 ± 16.0% for angular acceleration (Fig. 5). After salvaging, head acceleration events were re-run through the machine learning algorithm to determine if the salvaging process was successful. In 13 (62%) of head acceleration events that had originally been labeled decoupled, the salvaging process successfully converted the prediction to coupled. The remaining 8 (38%) were considered unsalvageable.

## Discussion

We have presented a machine learning model for detecting decoupling in instrumented mouthguard six-degree-of-freedom measurement signals. Our final model is intentionally low in feature count and complexity to avoid over-fitting the training dataset derived from laboratory head impact events. We valued sensitivity to detecting an actual decoupling event because the subsequent salvaging process minimally influenced peak kinematic accuracy in coupled events but considerably increased accuracy in decoupled events.

Relative to other decoupling prediction methods, our model had the highest sensitivity to decoupling in the training dataset. Although other methods had higher specificities, our model was the most balanced between sensitivity and specificity. In field head acceleration events, the Delta-prox method had the most similar decoupling prediction rate to our model. When considering all head acceleration events, however, the iMG manufacturer’s Quality prediction score was most similar to our model regarding agreement in both coupling status categories.

Our model differed from the other tested methods in that other methods use proximity sensor readings from the iMG, while our machine learning model does not. Our model uses information about known decoupling events [16] to detect decoupling. We used features from the kinematic signals rather than lower sampling rate proximity readings because coupling status can change throughout an impact event [14] and lower sampling rates may miss these changes. Therefore, when salvaging data is the goal, it is essential to classify coupling status with data from within the impact duration rather than only before and after. Additionally, infrared proximity sensors are fundamentally measuring different physical phenomena than the kinematic sensors upon which our presented algorithms rely. Proximity sensors measure the distance to a surface by calculating the intensity of reflected infrared light; these reflections can be influenced by factors other than true iMG motion relative to the dentition. The kinematic sensors, on the other hand, are measuring iMG motion directly, but here the challenge is to discern iMG motion from head impact characteristics, as we have attempted to train our model to do.

The supplementary coupling prediction methods we included had varying levels of agreement with one another. The modified Luke et al. method agreed well with the Delta-prox method in the laboratory training data. This agreement was likely due to both methods using only proximity readings as inputs and the controlled laboratory setting, which resulted in no iMG motion relative to the teeth except during impact. On the other hand, the Delta-prox method did not always agree with the Quality method. This lack of agreement likely comes from the manufacturer’s use of kinematic signal features in their Quality label assignment.

Advanced published post-processing methods such as HEADSport and AI-denoising offer similar promise to the methods presented: the ability to remove artifacts from iMG field data while keeping relevant head kinematic information. In those methods and ours, signal frequency content is used to decide how to move forward with a given impact most appropriately. Although the other advanced methods focus on signal-to-noise ratio and artifacts in general, we have chosen to focus on decoupling artifacts in particular as a problem with poor or loosely fitting iMGs. Issues with poor-fitting instrumented iMGs have been noted in sports, such as ice hockey [19], where mouthguard abuse is common, and helmet cages make ejecting an iMG mid-play inconsequential to players. Each of these advanced methods, including our presented algorithm and salvaging process, performs reasonably well in a laboratory environment, generally improving accuracy compared to a reference and removing egregious outliers. Testing the methods’ efficacy in the field is difficult, as coupling status is typically unknown, especially during an impact event.

Previously reported machine learning studies decided to reject data classified as non-impact or poor-quality [20, 21, 23]. To date, no study has reported methods for salvaging iMG data initially deemed to have decoupling artifacts. Our salvaging method improved accuracy in all three kinematics in laboratory decoupled impacts compared to the pre-salvaged iMG data. We applied our salvaging procedure to coupled laboratory head impacts to understand the potential effect this procedure could have should our algorithm misclassify in the field. The influence on peak kinematics was minimal for coupled data. This was expected because the salvaging procedure removes noise associated with decoupling, such as late, high-frequency peaks—these artifacts should not be present in coupled impacts. Peak angular acceleration was affected the most, with a higher percent difference in mean after the salvaging process. Despite the minimal effects, we do not recommend applying our salvaging methods to any impact data unless decoupling is suspected to be the primary noise source in the signal to ensure the greatest possible kinematic accuracy.

We expect salvaging field head acceleration events to have results similar to our laboratory results. Our preliminary sweep of the available field data reinforced this. We observed similar patterns of change from pre- to post-salvaging in our predicted-decoupled field data as we saw in our laboratory decoupled head impacts, with salvaged data having lower peak kinematics. After re-running the salvaged head acceleration events through our classification model, we found that 38% were unsalvageable. This likely means that decoupling was not the dominant source of noise in those 8 head acceleration events. Field data will inherently include more noise sources (e.g., chewing or jaw clack), but when decoupling is the primary source of noise in the signal, the presented salvaging methods can reduce kinematic error. Additional differences between laboratory head impacts and field head acceleration events include differences in rigidity between the NOCSAE headform surrogate and living volunteers and differences in neck response during head impact between surrogates and volunteers. Each of these factors may influence the accuracy and capability of our algorithms to successfully detect and remove decoupling artifacts.

Researchers should undertake impact salvaging on laboratory or field data with caution. Each recorded event has unique characteristics (e.g., head impact vs. body impact) that will influence the degree to which the presented machine learning model and salvaging process will apply. We recommend reviewing decoupled head impact signals in more detail to ensure they seem reasonable and salvageable. For this algorithm to be maximally effective, primary kinematic signals for linear acceleration should follow a half-sinusoid pattern and be approximately 3–7 ms from noise floor to peak, based on laboratory studies of head impacts [33, 34] (see Appendix A for example kinematic traces). If the observed primary kinematics from a field head acceleration event differ considerably from laboratory head impacts, the coupling prediction and salvaging results should be treated with lower confidence. One possible method for determining the success of the salvaging process is to re-run salvaged field head acceleration events through the presented decoupling-detection algorithm. If the algorithm still detects decoupling after salvaging and the signal pattern does not resemble a laboratory head impact, the signal may need to be discarded rather than salvaged. This algorithm was trained and developed using laboratory head impact data; therefore, applicability to field data where head contact is not the primary acceleration mechanism is limited.

In previously reported machine learning algorithms for rejecting non-impact signals, the authors suggested using some combination of proximity sensors, sensor thresholding, or both to pre-screen data before input to their models for further accuracy improvement [19–21, 35]. Likewise, we recommend a combination of methods for best removing decoupling artifacts: researchers should fit iMGs with the best obtainable coupling (e.g., custom fit); use a proximity-sensing method, such as Luke et al.’s; use an optimized data inclusion or trigger threshold [31, 36]; and use an artifact attenuation post-processing method such as HEADSport for unhelmeted sports or our decoupling-specific artifact attenuation method when helmets are worn and decoupling is common. A combination of these artifact mitigation and attenuation methods, along with following Consensus Head Acceleration Measurement Practices (CHAMP) recommendations [37, 38], could further inform which head acceleration events are of the highest quality. Salvaging should be considered a last resort to retain as much information as possible from lower-quality signals, and researchers should report what proportion of data had to be salvaged. When predicting artifact presence during an impact, we suggest that methods using the complete time series from six-degree-of-freedom measurement signals can best detect decoupling, as decoupling can happen during an impact event.

Our study has several limitations. We generated our machine learning model using training data from a single Prevent Biometrics boil-and-bite iMG in a laboratory test series. If decoupling could be reliably labeled in the field, these data would serve as a more diverse and relevant set of training data for similar algorithms in future. Additionally, because field data has more noise sources than a controlled laboratory environment, the coupling prediction and salvaging processes may have lower accuracy or applicability on field data than they did on the laboratory data. Future studies should further validate the presented methods, ideally with a labeled field impact dataset or, at minimum, human subjects in a laboratory environment. Another limitation is the fact that the iMG did not collect the primary impact for 32 rigid impacts. Although it is unclear why these impacts were not collected, we postulate that they could have been rejected due to the iMG’s firmware (version 2.0.14) or triggering thresholds [36]. Lastly, we would like to reiterate that we modified Luke et al.’s methods, which were developed initially using field data, to better comprehend our laboratory training data. Therefore, the accuracy of Luke’s original methods may not be reflected in our laboratory results section.

## Conclusion

This study contributes a novel machine learning algorithm to the field of head impact monitoring that enables the detection of iMG decoupling events in field head impacts. We also introduce an accurate method of salvaging kinematic data from most of these decoupling events. This study will help to enable faster and more accurate field studies of head impact exposure in volunteers, ultimately growing the field’s capability to answer important research questions about individual tolerance and head impact exposure.

## Figures and Tables

**Fig. 6:**
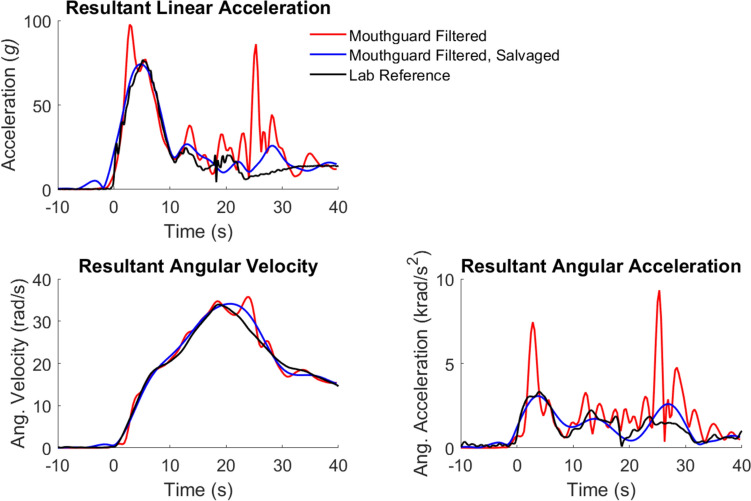
Example decoupled laboratory impact: 75-g padded rear impact in the 1.6-mm gap condition. Post-impact mouthguard data spikes were present in the original, filtered signals (red lines) but were removed or mitigated via salvaging (blue lines). Salvaging drastically reduced error relative to the reference measurement (black lines)

**Fig. 7:**
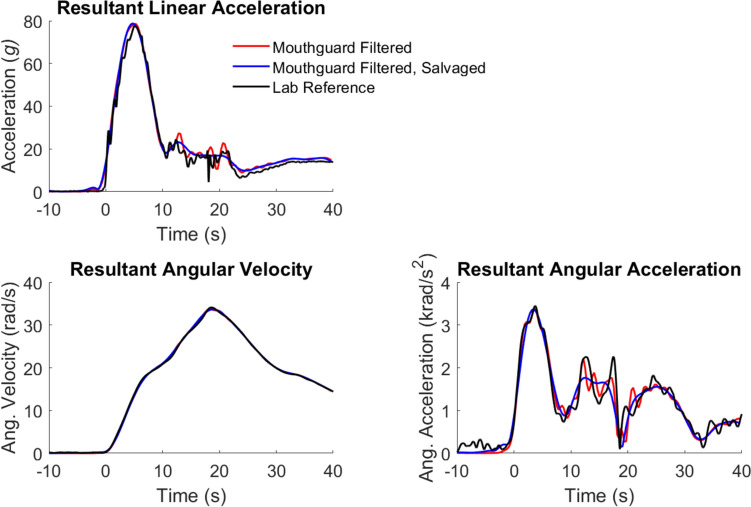
Example coupled laboratory impact: 75-g padded rear impact in the 0-mm gap condition. The salvaged signals (blue lines) were minimally affected compared to the original (red lines), showing that the salvaging process is generally safe to use on coupled head impacts in the case of a misclassification by the ML classification algorithm

**Fig. 8:**
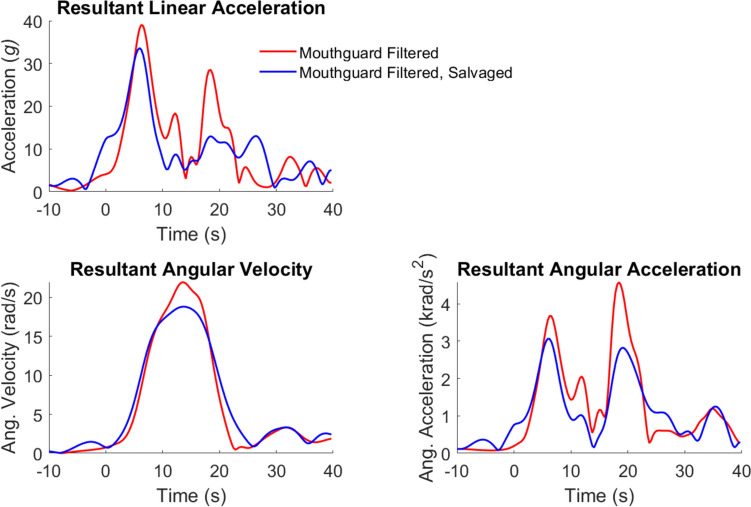
Example field impact predicted to have been decoupled by the ML model. Post-impact data spikes were present in the original, filtered signals (red lines) but were removed or mitigated via the salvaging process (blue lines)

**Fig. 9:**
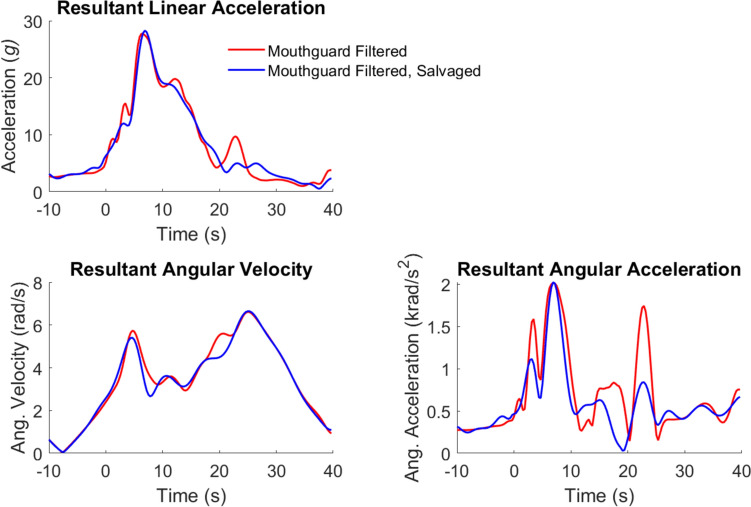
Example field impact predicted to have been coupled by the ML model. The salvaged signals (blue lines) were minimally affected compared to the original (red lines), and peak values are nearly identical

## Appendix: Example Kinematic Traces

### Laboratory Data

Decoupling was simulated in a laboratory test series by placing a boil-and-bite mouthguard on a headform dentition and leaving an open-mouth gap condition between the mouthguard and bottom dentition. Exemplar decoupled and coupled laboratory head impacts are presented, showing both the error created by gap conditions and the effect of the salvaging process on the mouthguard signals (Figs. 6 and 7).

## Field Data

We also used our developed machine learning classification algorithm to predict the status of field head acceleration events. Coupled head acceleration events were affected less by the salvaging process than those predicted to have decoupled, which agreed with the laboratory analysis (Figs. 8 and 9).
